# “Trust your gut”: exploring the connection between gut microbiome dysbiosis and the advancement of Metabolic Associated Steatosis Liver Disease (MASLD)/Metabolic Associated Steatohepatitis (MASH): a systematic review of animal and human studies

**DOI:** 10.3389/fnut.2025.1637071

**Published:** 2025-09-10

**Authors:** Wesam Bahitham, Yusra Banoun, Mutep Aljahdali, Ghufran Almuaiqly, Shahad M. Bahshwan, Linah Aljahdali, Faisal M. Sanai, Alexandre S. Rosado, Consolato M. Sergi

**Affiliations:** ^1^King Abdullah International Medical Research Center-WR, King Saud bin Abdulaziz University for Health Sciences, Ministry of National Guard for Health Affairs, Jeddah, Saudi Arabia; ^2^Bioscience, Biological and Environmental Sciences and Engineering Division, King Abdullah University of Science and Technology, Thuwal, Saudi Arabia; ^3^Department of Pharmacy Practices, College of Pharmacy, Umm Al-Qura University, Makkah, Saudi Arabia; ^4^Department of Clinical Biochemistry, Faculty of Medicine, King Abdulaziz University, Jeddah, Saudi Arabia; ^5^Gastroenterology Unit, Department of Medicine, King Abdulaziz Medical City, Jeddah, Saudi Arabia; ^6^Anatomic Pathology Division, Children’s Hospital of Eastern Ontario, University of Ottawa, Ottawa, ON, Canada; ^7^Department of Laboratory Medicine and Pathology, University of Alberta, Edmonton, AB, Canada

**Keywords:** NAFLD, MASLD, NASH, MASH, metabolic syndrome, gut, microbiome, dysbiosis

## Abstract

Metabolic Associated Steatosis Liver Disease (MASLD) and its advanced form, Metabolic Associated Steatohepatitis (MASH), represent growing global health concerns closely linked to obesity, type 2 diabetes mellitus (T2DM), and metabolic syndrome. The gut microbiome has emerged as a key modulator in MASLD pathogenesis through the gut–liver axis, influencing hepatic fat accumulation, inflammation, and fibrosis via microbial metabolites and immune responses. Dysbiosis–characterized by altered microbial diversity and composition–contributes to hepatic lipid dysregulation, systemic inflammation, and impaired bile acid signaling. Metabolites such as short-chain fatty acids (SCFAs), trimethylamine-N-oxide (TMAO), and ethanol play critical roles in disease progression. Recent innovations in precision medicine, including microbiome profiling, metabolomics, and genomics, offer promising diagnostic and therapeutic strategies. Targeted probiotics, fecal microbiota transplantation (FMT), and personalized dietary interventions are under investigation for modulating the gut microbiome. This systematic review, conducted in accordance with PRISMA 2020 guidelines, is the first to comprehensively integrate both animal and human studies on MASLD/MASH-related gut microbiome alterations. It uniquely synthesizes microbial taxa, functional metabolites, and region-specific patterns–including data from underrepresented MENA populations. Eligible studies from PubMed, Scopus, and Web of Science evaluated microbial composition, metabolite profiles, and associations with steatosis, inflammation, and fibrosis. The findings underscore the diagnostic and therapeutic potential of microbiome modulation and emphasize the need for longitudinal, mechanistically driven studies. This systematic review is the first to integrate both animal and human studies on MASLD/MASH-related gut microbiome alterations. Unlike previous reviews, it uniquely emphasizes microbial taxa, functional metabolites, and region-specific patterns, including underrepresented MENA populations. By synthesizing findings from diverse cohorts, this review highlights diagnostic and therapeutic opportunities while identifying persistent gaps in longitudinal data, regional representation, and multi-omics integration.

## Introduction

Metabolic Dysfunction-Associated Steatotic Liver Disease (MASLD) has become a common concern in public health, impacting around 25%–30% of adults worldwide (1, 2). According to World Health Organization (WHO) estimates, the growing prevalence of MASLD reflects the rising trends in obesity and type 2 diabetes mellitus (T2DM) worldwide, highlighting the disease’s importance on a global health scale. MASLD is characterized by the accumulation of fat in the liver without heavy alcohol consumption and can range from simple hepatic steatosis to more severe forms such as Metabolic Dysfunction-Associated Steatohepatitis (MASH) fibrosis, cirrhosis, and even hepatocellular carcinoma (HCC) (2, 3). The increasing incidence of MASLD underscores the urgent need for public health measures because it has become the most common chronic liver condition worldwide, paralleling trends in obesity and T2DM that negatively affect metabolic health (4). The widespread presence of MASLD can be seen in its high rates in different regions, each influenced by lifestyle, diet, and metabolic health. In Western Europe and North America, high rates of obesity and metabolic syndrome contribute to a prevalence of approximately 25%–30% among adults (2). In the United Kingdom, the prevalence of MASLD has increased due to obesity and metabolic syndrome, leading to higher rates of MASH, a more severe form of the condition that could result in liver failure. In North America, MASLD is a primary reason for liver transplants, stressing the critical necessity for action. The Middle East and North Africa (MENA) region is experiencing alarmingly high rates of MASLD, primarily driven by the increasing prevalence of obesity and T2DM. The prevalence of MASLD in Saudi Arabia is projected to increase from 25.8% in 2017 to 31.7% by 2030, reflecting a 48% rise in the number of cases, reaching an estimated 12.5 million individuals. Similarly, in the United Arab Emirates (UAE), the prevalence is anticipated to reach 30.2% by 2030, corresponding to a 46% increase in cases, totaling approximately 372,000. These trends highlight the critical need for targeted public health interventions to address the region’s escalating metabolic health burden (5).

In East Asia, there is a growing occurrence of metabolic disorders due to urbanization and changes in diet, resulting in increased prevalence rates. In China, around 29.2% of adults are affected by MASLD, while in South Korea, the rate is approximately 30%, and younger populations in Japan are experiencing a higher incidence than in the past (1). In South Asia, especially India, MASLD rates reflect these trends because of the high occurrence of T2DM and changing eating habits (1, 4). These worldwide trends emphasize the immediate necessity for local measures to control the disease’s rise and lessen its effects on public health. It is crucial to address metabolic health, dietary habits, and obesity through specific strategies tailored to each region to lessen the impact of MASLD on healthcare systems globally. Emerging evidence suggests that artificial sweeteners may influence gut microbiota composition, with potential metabolic and inflammatory consequences relevant to MASLD progression. Recent evaluations by IARC/WHO have also raised questions regarding long-term health effects, including carcinogenicity, which warrant further investigation in the context of liver disease (6, 7).

These regional and global trends in MASLD prevalence are closely intertwined with rising rates of metabolic disorders–particularly type 2 diabetes mellitus (T2DM), which shares overlapping pathophysiological pathways with liver steatosis. The connection between MASLD and T2DM highlights a reciprocal relationship (8). Nearly 70% of individuals with T2DM are estimated to also have MASLD, where insulin resistance (IR) and hyperglycemia worsen hepatic fat accumulation, while MASLD contributes to systemic IR and pancreatic β-cell dysfunction, increasing the risk of T2DM (9, 10). However, not all individuals with T2DM develop MASLD, and not all MASLD patients progress to T2DM. Still, their coexistence significantly amplifies the risk of complications such as fibrosis and cirrhosis (8, 11).

Beyond traditional metabolic risk factors, growing attention has turned to the gut microbiome as a central modulator of MASLD through its influence on hepatic inflammation, lipid metabolism, and systemic insulin resistance. The gut microbiome is being increasingly acknowledged as a significant contributor to the development of MASLD, with the gut-liver axis becoming a key mechanism. Dysbiosis (microbial imbalance) in the gut microbiota can result in liver inflammation (hepatitis or “portitis”), fibrosis, and other metabolic disturbances. Understanding these fundamental processes is essential for the development of effective microbiome-targeted therapies. Recent research indicates that altering the composition of gut bacteria can improve liver health, suggesting that modulating the microbiome may serve as a promising therapeutic strategy for managing MASLD and MASH (11–14).

The gut microbiome is mechanistically relevant to MASLD due to its direct and indirect interactions with hepatic metabolism. Gut-derived bacterial metabolites–including SCFAs, ethanol, ammonia, and trimethylamine-N-oxide (TMAO)–influence hepatic fat accumulation, insulin sensitivity, and inflammatory signaling. Dysbiosis also disrupts gut barrier integrity, leading to increased translocation of microbial components such as lipopolysaccharide (LPS) into the portal circulation, which promotes hepatic inflammation and fibrosis. Through the gut–liver axis, these mechanisms link intestinal microbial composition to the pathogenesis and progression of MASLD. The specific mechanisms by which the gut microbiome influences liver disease pathogenesis are described in detail in the sections below.

To address the growing burden of MASLD, comprehensive global public health strategies are required. These should include lifestyle changes, genetic screening, and microbiome-focused treatments. Region-specific interventions tailored to local dietary habits, lifestyle factors, and genetic predispositions are essential to prevent the rapid increase of MASLD and to reduce its impact on global health (15, 16). By implementing these strategies through coordinated efforts, the healthcare community can better manage the escalating public health issue, improve individual outcomes, and alleviate the strain on healthcare systems worldwide (17).

This systematic review aims to comprehensively evaluate current evidence on the role of gut microbiome dysbiosis in MASLD/MASH pathogenesis, integrating findings from animal models and clinical studies. Detailed insights into how gut dysbiosis may contribute to liver inflammation and fibrosis are vital for developing future microbiome-targeted treatments, highlighting the importance of integrating these insights into therapeutic strategies. Despite the rapidly rising prevalence of MASLD in MENA countries, few microbiome-focused reviews have addressed regional microbial patterns, dietary drivers, or therapeutic opportunities in these populations. Furthermore, existing reviews often separate animal and human findings. To bridge this gap, our systematic review integrates both preclinical and clinical studies, demonstrates MENA-specific insights, and explores microbial function and metabolite pathways relevant to MASLD progression.

### Gut microbiome dysbiosis in MASLD/MASH

The gut microbiota is diverse, consisting of various bacterial species classified by genus, family, order, and phyla. The composition of an individual’s gut microbiota differs from person to person. It is shaped early in life and influenced by factors such as birth gestation, type of delivery, feeding methods, and weaning. External factors like antibiotic use also play a role. Despite this variability, most healthy adults share a core set of bacterial species. For instance, *Escherichia coli* is commonly found in many individuals. The dominant bacterial groups in the adult gut are Bacteroidetes and Firmicutes, while Actinobacteria, Proteobacteria, and Verrucomicrobia are present in smaller amounts. In addition to bacteria, the gut also contains methanogenic archaea (mainly *Methanobrevibacter smithii*), eukaryotes (primarily yeasts), and viruses (mostly bacteriophages) (18, 19). Although these elements are consistently found in the gut, identifying a core set of species-level phylotypes has revealed as standard microbes such as *Faecalibacterium prausnitzii*, *Roseburia intestinalis*, and *Bacteroides uniformis*. However, these species may comprise less than 0.5% of the microbial population in some individuals (20, 21). Several factors can negatively impact the beneficial gut flora, including antibiotic use, psychological and physical stress, radiation, changes in gastrointestinal (GIT) peristalsis, and dietary modifications (22). The balance of gut microbiota is influenced by various host factors such as lifestyle, diet, medications, hygiene, health, and genetics. This imbalance, or dysbiosis, may contribute to the onset of immune, metabolic, neurodegenerative, psychological, and other infectious diseases, including “long COVID-19” (23–26).

**FIGURE 1:**
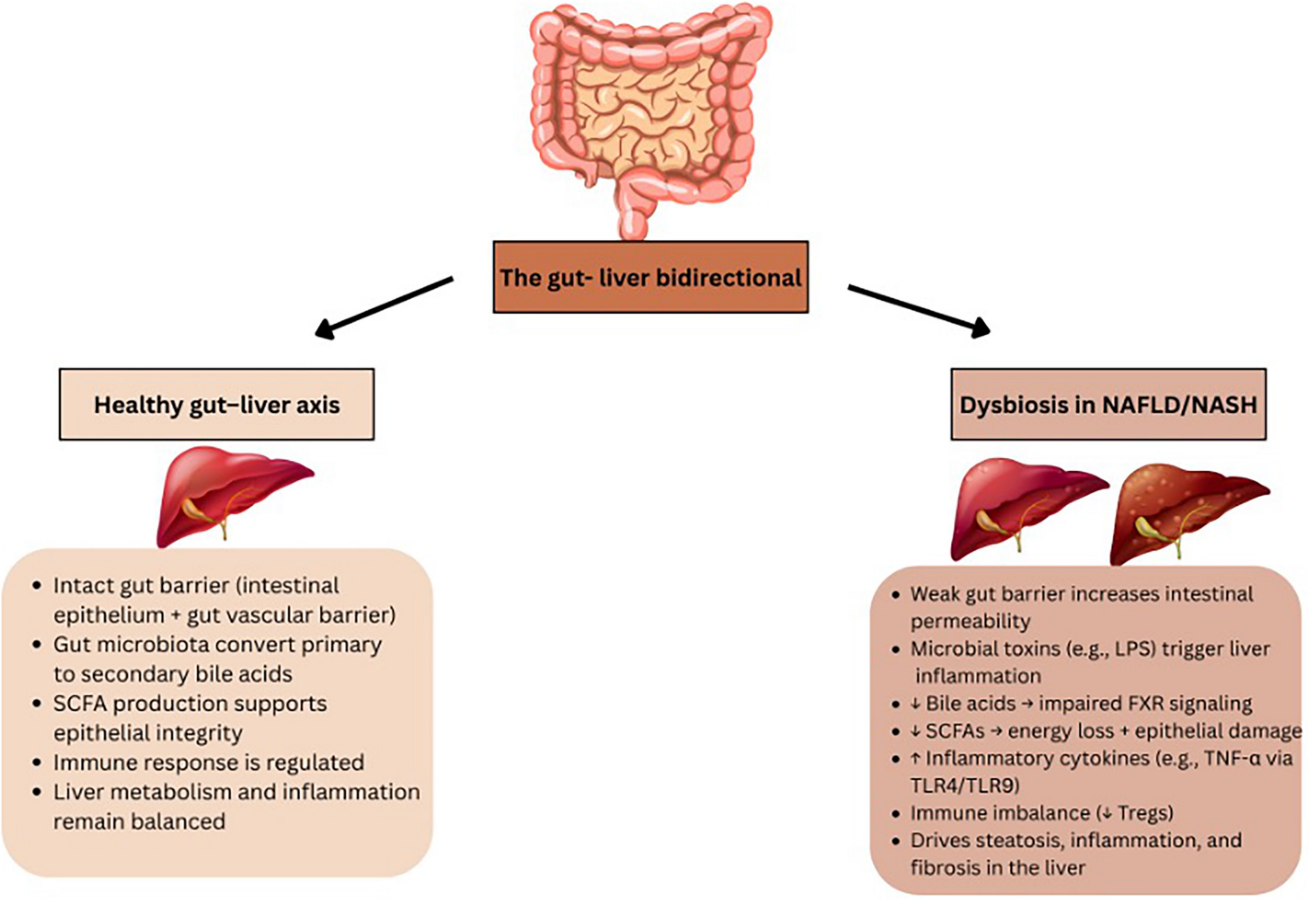
Overview of the gut–liver axis in health and disease. The healthy gut–liver axis involves a stable gut barrier, regulated immune responses, and microbial production of SCFAs and bile acid metabolism, which together support liver homeostasis. In contrast, dysbiosis in NAFLD/NASH leads to barrier dysfunction, increased intestinal permeability, translocation of microbial products (e.g., LPS), decreased SCFA and bile acid production, immune imbalance, and activation of pro-inflammatory pathways, ultimately promoting hepatic steatosis, inflammation, and fibrosis.

The liver and microbiome interact closely via the portal vein, which transports gut-derived substances to the liver, while bile acids and antibodies from the liver provide feedback to the intestine. Bile acids engage with nuclear receptors to regulate metabolism and play a key role in controlling gut microbiota. This interaction occurs at the gut mucosal barrier, where intestinal epithelial cells maintain gut balance by keeping gut microbiota separate from the host’s immune cells (27) (Figure 1). Figure 1 presents the structural organization of the intestinal barrier and illustrates how its disruption contributes to the development and progression of MASLD and MASH through increased microbial translocation and systemic inflammation. The interface between the liver and the microbiome is the gut mucosal barrier, which is made up of intestinal epithelial cells that maintain gut homeostasis by segregating gut microbiota and host immune cells. The mucus barrier and diet influence microbiota composition, impacting health by preventing harmful microbiota-epithelium contact that could trigger inflammation. The barrier also nourishes and stabilizes microbiota, helping them remain despite digestive movement. A gut vascular barrier (GVB) blocks bacteria from entering the portal circulation and reaching the liver. However, certain pathogenic bacteria and possibly some pathobionts have developed ways to bypass this barrier. This barrier may become compromised in some pathological conditions, such as MASLD and MASH. The microbiota shapes the entire intestinal barrier through the coordinated activity of structural components (such as mucus and epithelial cells), immune cells (including intraepithelial and lamina propria cells), and soluble mediators (like IgA and antimicrobial peptides). Alterations in any of these elements can disrupt the intestinal barrier. Additionally, the microbiota can influence the effectiveness of treatments by interacting with the immune system (27). Communication between the gut and liver occurs through bile acids, with the liver producing bile that shapes the gut microbiome, while gut microbiota modulates bile acid composition. The gut-liver axis, the bidirectional interaction, is essential for maintaining physiological health. In conditions like MASLD, which ranges from simple fatty liver to more severe stages like MASH with fibrosis, gut dysbiosis plays a significant role. Factors like obesity, diet, and metabolic syndrome contribute to changes in the gut microbiota, increasing intestinal permeability and allowing harmful metabolites such as lipopolysaccharide (LPS) to enter the liver, worsening liver injury. Microbial metabolites like ethanol, phenylacetate, and TMAVA are linked to the progression of MASLD, while metabolites derived from tryptophan may help reduce liver inflammation. Additionally, patients with MASLD or MASH often exhibit altered bile acid profiles, disrupting liver function (28) (Figure 1).

Bile acids play a crucial role in shaping the gut microbiota through a bidirectional interaction, where the microbiota influences bile acid metabolism, and bile acids, in turn, affect the microbiota. After being converted into secondary bile acids, they signal through the farnesoid X receptor (FXR) in the intestinal epithelium, enhancing the epithelial barrier, repairing damage to the gut vascular barrier, and regulating aspects of metabolic syndrome. However, studies in mice with FXR knockouts have yielded mixed results, indicating that FXR may have different roles in the development of NASH (27). The Pathogen-associated molecular patterns (PAMPs) play a role in liver damage in MASLD. Inflammasome deficiency-induced changes in the gut microbiota lead to hepatic steatosis and inflammation through the portal circulation, triggering toll-like receptors (TLRs) such as TLR4 and TLR9 agonists. This process increases necrosis factor-alpha (TNF-α) production in the liver, intensifying inflammation. Postbiotics, such as short-chain fatty acids (SCFAs) like acetate, propionate, and butyrate, are produced during the breakdown of dietary fibers. These substances affect the intestinal epithelial barrier, immune system, and microbiota. SCFAs, in particular, influence immune cell differentiation, including T regulatory cells, and enhance the microbicidal activity of macrophages. Additionally, postbiotics can impact the balance between brown and white adipose tissue. The thickness and integrity of the mucus barrier, which covers the intestinal epithelium, can vary depending on the gut segment (27).

## Methods

**FIGURE 2:**
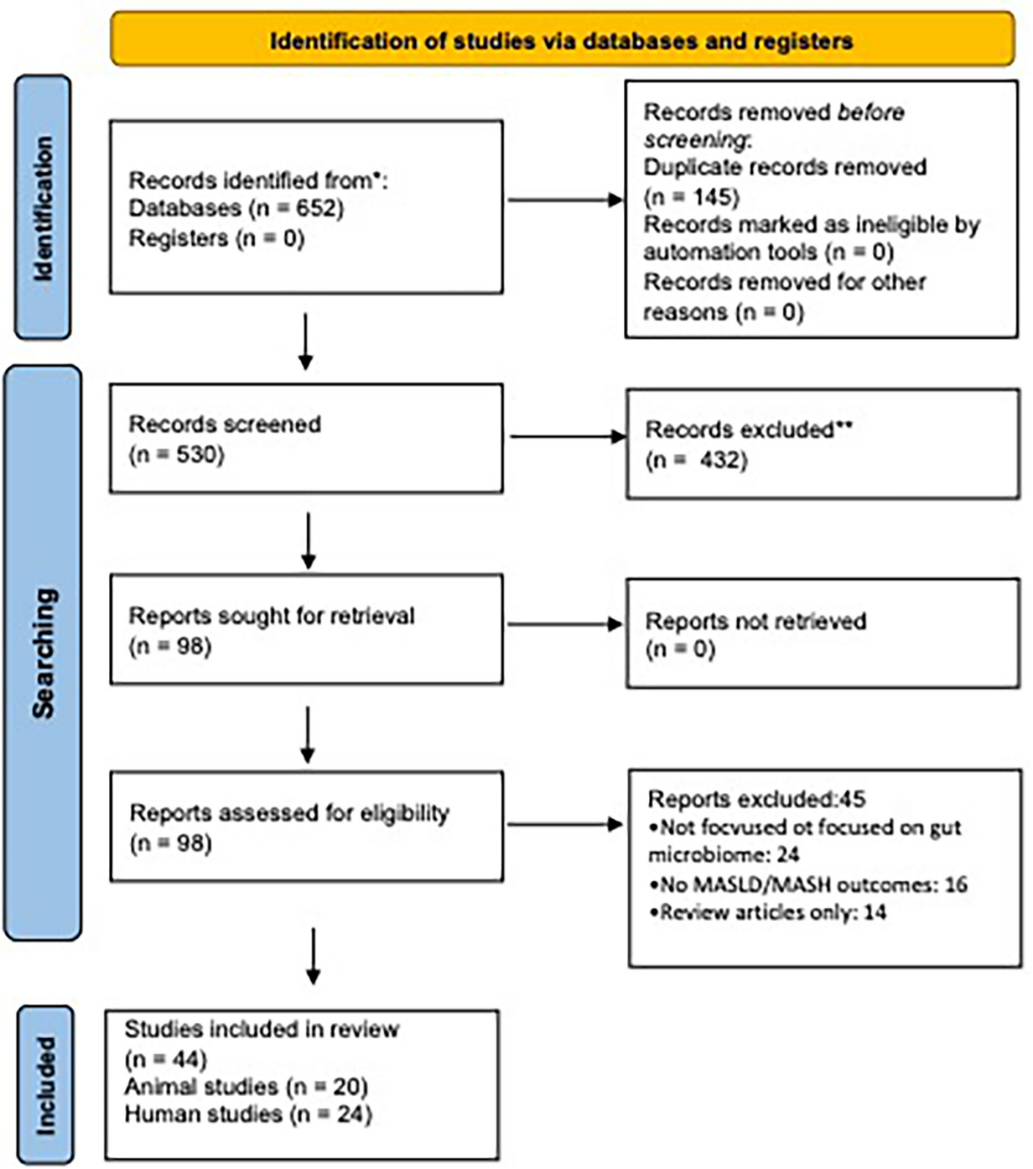
PRISMA 2020 flow diagram. PRISMA 2020 flow diagram outlining the study identification, screening, eligibility assessment, and inclusion process for this systematic review. A total of 92 studies were included in the final analysis following exclusion of duplicates and irrelevant records.

We conducted a systematic literature search in PubMed, Scopus, and Web of Science databases up to March 2025. Keywords included “MASLD,” “MASH,” “gut microbiota,” “microbiome dysbiosis,” “gut-liver axis,” “microbial metabolites,” “animal models,” and “human studies.” Boolean operators and MeSH terms were used where applicable. As shown in Figure 2, the study selection process followed the PRISMA 2020 guidelines, detailing records identified, screened, and included in the final analysis.

### Registration and protocol

This review was not registered in a public database such as PROSPERO.

#### Inclusion criteria

- Original studies (animal or human) examining the role of gut microbiota in MASLD/MASH.
- Studies reporting gut microbiome composition, metabolite profiling, or intervention outcomes.
- Peer-reviewed articles in English.

#### Exclusion criteria

- Editorials, conference abstracts, case reports without mechanistic data.
- Studies focusing exclusively on viral hepatitis or alcohol-related liver disease.

#### Search strategy

The search strategy included the following terms: (“MASLD” OR “Metabolic Associated Steatosis Liver Disease” OR “MASH” OR “Metabolic Associated Steatohepatitis” OR “NAFLD”) AND (“gut microbiota” or “microbiome” OR “dysbiosis” OR “gut-liver axis” OR “microbial metabolites” OR “animal models” OR “human studies).

#### Study selection

Titles and abstracts were screened independently by two reviewers. Full texts of potentially eligible studies were assessed for inclusion. Discrepancies were resolved through (29).

#### Data extraction and synthesis

Data were extracted using a standardized form, capturing: study design, model used, microbiome changes, metabolites involved, and liver-related outcomes. Findings were synthesized narratively and tabulated for clarity.

Despite the growing number of studies examining the gut microbiome in MASLD, the majority of included human research was cross-sectional. This limits our ability to understand temporal microbial shifts or determine causality in disease progression. Longitudinal studies–particularly those that evaluate microbiome dynamics in relation to steatosis, fibrosis, or clinical interventions–remain sparse. The lack of prospective data remains a key gap in the field, constraining the development of microbiome-based diagnostics or therapeutic monitoring tools.

#### Risk of bias assessment

Risk of bias was not formally assessed in this narrative synthesis. Included studies were appraised based on study design, sample size, and relevance to the research objectives.

#### PRISMA flow diagram

(Reference the flowchart: A PRISMA flow diagram illustrating the study selection process is presented in Figure 2).

## Results: evidence linking dysbiosis to the progression of MASLD/MASH

### Animal studies

Animal studies have played a crucial role in clarifying the mechanisms by which gut microbiota influence the progression of MASLD/MASH. These studies frequently employs to replicate human disease conditions, offering valuable insights into the impact of changes in gut microbiota on liver health. The relationship between observations in animal models and human patients is essential. However, animal models can replicate specific features of human disease, they also possess limitations that need to be recognized.

Animal models are essential for comprehending the pathophysiology of diseases such as MASLD/MASH. The classification of these models includes induced and spontaneous types. Induced models arise from targeted interventions, whereas spontaneous models develop naturally within specific strains (30, 31). For instance, rodents are often utilized because of their genetic and physiological similarities to humans, enabling the observation of the effects of gut microbiota changes on liver health (32). Studies show that changes in gut microbiota can result in heightened intestinal permeability, allowing LPS to enter the bloodstream, which initiates inflammation and plays a role in liver damage (33). This mechanism has been documented in numerous animal studies, underscoring the promise of microbiota-targeted therapies for addressing MASLD/MASH.

Nonetheless, although these animal models offer significant insights, it is crucial to acknowledge their constraints. Variations in metabolic processes, anatomical structures, and the progression of diseases between animals and humans can influence how findings can be applied across species. For instance, specific therapeutic interventions that show efficacy in animal models might not produce comparable outcomes in human clinical trials because of these discrepancies (31, 32). A thorough assessment of how animal model findings relate to human conditions is essential for progressing therapeutic approaches. The subsequent sections will explore the use of various animal models in gut microbiome research, as outlined in Tables 1A, B.

**TABLE 1A:** Dietary, genetic, and gnotobiotic models of MASLD/MASH: commonly used dietary, genetic, and gnotobiotic animal models in MASLD and MASH research.

Animal model	MASLD/MASH associations with certain gut microbiomes	Common applications	Advantages	Disadvantages	Relevance to human pathology
Germ-free mice	Germ-free mice exhibit vulnerability to MASLD upon colonization with certain bacteria, which can provoke steatosis and inflammation (36, 112).	This model was used to investigate microbial contributions to hepatic health and to evaluate probiotics (112, 113).	Offers an explicit understanding of microbial functions in a regulated setting (112, 114).	The deficiency in immune system complexity may not precisely represent human microbiome interactions (36, 115).	Provides a fundamental comprehension of the impact of particular microorganisms on liver health (36, 116, 117).
C57BL/6 mice	These mice exhibit obesity and steatosis when subjected to high-fat diets (HFDs), accompanied by altered gut microbiota associated with liver inflammation (114, 115).	Utilized for nutritional interventions, metabolic studies, and pharmacological testing (112, 113).	Well-defined metabolic pathways are extensively utilized in obesity studies (114, 115).	May not wholly emulate human metabolic reactions; genetic diversity may influence results (113, 114).	Accurately replicates human dietary effects and metabolic syndrome (70, 112, 113).
db/db mice	Hyperglycemia in db/db mice is associated with elevated liver fat; dysbiosis is connected to worsened liver injury (112, 113).	Beneficial for research on metabolic syndrome and studies of insulin resistance (114, 115).	Genetic susceptibility contributes to the comprehension of obesity-related liver illness (112, 113).	Constrained by genetic background; may not encompass all aspects of human MASLD/MASH (114, 115).	Significant for examining the relationships between obesity-related non-alcoholic steatohepatitis (MASH) and diabetes (36, 112).
Methionine-choline deficient diet (MCD) models	Induces steatosis that mimics MASH pathogenesis; certain gut bacteria could influence disease severity (114, 115).	Examining the effects of nutrition on liver pathology and the efficacy of pharmacological trials (112, 113).	Directly simulates MASH pathogenesis and promotes investigations on dietary manipulation (114, 115).	Less relevant for extensive dietary situations and may not accurately represent intricate human diets (36, 112).	Directly pertinent to human MASH contexts and dietary impacts on hepatic health (114, 115).
High-fat high-fructose diet models	Contemporary eating trends adversely influence liver function; dysbiosis is associated with heightened hepatic fat accumulation and inflammation (112, 113).	Investigating the effects of lifestyle on MASLD/MASH and evaluating nutritional treatments (114, 115).	It illustrates modern dietary practices, which are valuable for analyzing lifestyle-associated disorders (112, 113).	May not encompass all dimensions of human dietary variability; constrained by particular dietary formulations (112, 114).	Offers insights on lifestyle-associated MASLD/MASH and their public health ramifications (36, 115).
Zucker fatty rats	Demonstrates obesity and insulin resistance; alterations in gut microbiota are associated with hepatic fat storage and inflammation (112, 113).	Longitudinal investigations of metabolic disorders and the evaluation of treatment strategies (114, 115).	A naturally occurring obesity model is pertinent for investigating metabolic syndrome’s consequences on hepatic health (112, 113).	Restricted genetic variety relative to alternative models; may not adequately reflect human situations (36, 115).	Beneficial for comprehending genetic susceptibility in the progression of MASLD/MASH (112, 114).

These models simulate core features such as steatosis, insulin resistance, and inflammation through nutritional interventions, inherited traits, or germ-free conditions. Typical durations and measured endpoints (e.g., steatosis, ALT/AST, cytokines, insulin resistance markers, microbiota composition) are described in the animal models section for each model type, including HFD (8–16 weeks) and MCD (4–6 weeks) diets.

**TABLE 1B:** Translational and pharmacological models of MASLD/MASH: commonly used translational and pharmacological animal models, including Sprague-Dawley rats and non-human primates, that offer physiological relevance and experimental flexibility for simulating MASLD/MASH features and evaluating interventions.

Animal model	MASLD/MASH associations with certain gut microbiomes	Common applications	Advantages	Disadvantages	Relevance to human pathology
Sprague-Dawley rats	Fatty liver develops under high-fat or MCD diets; modifications in gut microbiota correlate with the severity of liver injury (112, 113).	Pharmacological investigations and dietary treatments in MASLD/MASH models (114, 115).	A versatile model for many experimental situations with proven techniques accessible (112, 113).	Variability in reactions from environmental factors or dietary composition may obfuscate results (36, 115).	Pertinent for translational research connecting animal discoveries to human problems (112, 114).
Non-human primates	Human physiology and metabolism are closely replicated; alterations in gut microbiota are associated with the course of diet-induced fatty liver disease (36, 112).	Translational research for human-like reactions to dietary modifications or interventions (113, 115).	Significant importance owing to physiological parallels with humans, facilitating improved modeling of intricate interactions (36, 112).	Ethical constraints restrict utilization, substantial expenses, and logistical difficulties in maintenance (113, 115).	Offers essential insights into the translational dimensions of MASLD/MASH therapy for people (36, 112).

Typical durations and measured endpoints (e.g., steatosis, ALT, TNF-α, microbiota) are discussed in the main animal models section for each model type.

### Studies on germ-free (GF) mice

Germ-free mice have played a crucial role in clarifying the influence of gut microbiota on liver pathology related to MASLD/MASH. GF mice that are colonized with microbiota from patients with obesity or MASLD show a greater accumulation of hepatic fat and inflammation when compared to those that remain uncolonized. These finidings indicate the microbial communities can directly impact liver disease. For example, specific strains of Firmicutes or Bacteroidetes have demonstrated an ability to worsen steatosis and inflammation in these models (34–36). Moreover, fecal microbial transplantation (FMT) studies have validated this connection. GF mice that were given microbiota from MASH patients show hepatic steatosis and inflammatory changes, underscoring the causal role of gut microbiota in the development of MASLD (35–37). The results indicate that changes in gut microbiota composition are not just linked to MASLD/MASH but could play a significant role in its progression (Tables 1A, B).

### High-fat diet (HFD) models

High-fat diet models are extensively utilized to investigate MASLD/MASH because they effectively replicate the metabolic characteristics of the disease. Providing rodents with diets rich in fat (usually comprising 45%–75% of total caloric intake) results in notable alterations in gut microbiota composition, which is associated with elevated hepatic lipids and insulin resistance. For instance, C57BL/6 mice subjected to high-fat diets exhibit obesity and demonstrate increased levels of pro-inflammatory cytokines like TNF-α and interleukin-6 (IL-6), which are associated with liver inflammation and damage (35, 37). The dysbiosis caused by high-fat diets is marked by a reduction in beneficial bacteria such as Lactobacillus and a rise in pathogenic bacteria like *Escherichia coli*. This change plays a role in metabolic endotoxemia and liver inflammation, strengthening the link between gut microbiota and the advancement of liver disease (34, 36). HFD models typically require 8–16 weeks of dietary intervention to induce hepatic steatosis, inflammation, and early fibrosis. Common readouts include histological grading of steatosis, serum liver enzymes (ALT, AST), inflammatory cytokines (e.g., TNF-α, IL-6), insulin resistance indices, and gut microbiota profiling via 16S rRNA sequencing or metagenomics. Research shows that microbial signatures linked to high-fat diets can increase intestinal permeability, facilitating bacterial translocation that worsens liver inflammation (Tables 1A, B) (37, 38).

### Models of methionine-choline deficient diet (MCD)

Methionine-choline deficient diet is a prominent model for the swift induction of MASH in rodent studies. Mice on this diet exhibit notable hepatic inflammation and fibrosis within weeks, primarily attributed to disrupted lipid metabolism caused by a lack of choline. Research indicates that MCD feeding results in significant changes in gut microbiota composition, significantly an increase in Proteobacteria, which correlates with inflammatory responses and worsens liver pathology (39, 40). The MCD model shows a notable rise in pro-inflammatory cytokines like IL-1β and TNF-α in the intestines, suggesting a connection between gut inflammation and the advancement of liver disease (40, 41). MCD diet models generally run for 4–6 weeks and produce rapid-onset fibrosis and inflammation, with outcomes including liver histopathology, metabolic profiles, and microbial community composition. These duration ranges and endpoints are representative of standard MASLD/MASH experimental protocols. The swift emergence of MASH-like characteristics facilitates the investigation of early treatment options and underscores the influence of gut microbiota on liver inflammation modulation (Tables 1A, B).

#### Genetic models

Genetically modified models, such as the *db/db* and *ob/ob* mice, serve as essential tools for elucidating the progression of MASLD and MASH, given their inherent metabolic disturbances including obesity and insulin resistance. When subjected to dietary challenges, such as high-fat or high-fructose regimens, these models exhibit exacerbated hepatic pathology alongside pronounced alterations in gut microbiota composition. For example, *ob/ob* mice exposed to a high-fructose diet develop marked hepatic steatosis, which is paralleled by shifts in gut microbial populations that promote inflammatory pathways (42). These findings underscore the synergistic relationship between genetic susceptibility and environmental influences in shaping the gut-liver axis, ultimately contributing to liver disease progression (Tables 1A, B).

#### Combination models

Models that integrate genetic predisposition with dietary interventions provide a more comprehensive framework for studying MASLD/MASH pathogenesis. The KK-Ay mouse model, which possesses a genetic inclination toward obesity and type 2 diabetes, demonstrates severe hepatic injury and microbiota dysbiosis when challenged with a high-fat diet (39). Notably, these combination models more accurately replicate the multifactorial nature of human disease, revealing intricate interactions between gut microbial dysbiosis, compromised intestinal barrier function, and systemic inflammation (41). Such models provide valuable insights into the complex metabolic networks and inflammatory pathways that underlie disease progression, thereby highlighting potential therapeutic targets (Tables 1A, B).

The included animal models reflect various mechanisms by which gut microbiome alterations can influence hepatic steatosis, inflammation, and fibrosis in MASLD and MASH. A detailed comparison is provided in (Tables 1A, B). Typical durations for these models range from 4 to 6 weeks in MCD and germ-free studies to 8–16 weeks in HFD or genetic models. Across studies, key parameters measured include liver steatosis (via histology), serum liver enzymes (ALT/AST), pro-inflammatory cytokines (e.g., TNF-α, IL-6), insulin resistance markers, and gut microbial composition. By clarifying key pathophysiological mechanisms, they not only enhance our understanding of the gut-liver axis but also serve as indispensable platforms for the identification and validation of novel therapeutic strategies. Continued research leveraging these models will be vital for advancing translational efforts aimed at mitigating the global burden of liver disease.

While rodent models such as high-fat diet (HFD)-fed mice have been instrumental in uncovering microbiome–liver interactions, they do not fully recapitulate the complexity of human MASLD/MASH pathophysiology. Differences in diet, bile acid metabolism, immune responses, and microbial ecosystems between mice and humans pose translational challenges. To bridge this gap, future studies may benefit from incorporating humanized mouse models–such as germ-free mice colonized with human microbiota–as well as integrating multi-omics approaches (e.g., metagenomics, metabolomics, transcriptomics) to capture host–microbiome interactions more holistically. These strategies can improve the relevance of preclinical findings and enhance their applicability to human MASLD progression and treatment.

### Human studies

#### Altered microbial taxa in MASLD and MASH

Recent studies have thoroughly examined the connection between gut microbiota and MASLD. Rau et al. identified distinct gut microbial profiles in individuals with MASH, characterized by an abundance of SCFA-producing bacteria like *Fusobacterium* and *Prevotella*. Elevated concentrations of fecal short-chain fatty acids, especially acetate and propionate, correlated with enhanced pro-inflammatory immune responses, marked by an increased Th17/rTreg cell ratio. The results underscore the importance of microbial metabolites in managing low-grade inflammation, which contributes to liver inflammation and fibrosis, thus positioning the gut-liver axis as a crucial factor in the advancement of MASLD (43).

Recent studies have explored the connection between gut microbiota and MASLD. Rau et al. (43) identified distinct microbial profiles in individuals with NASH, marked by an abundance of SCFA-producing bacteria such as *Fusobacterium* and *Prevotella*. Elevated levels of short-chain fatty acids, particularly acetate and propionate, were linked to increased pro-inflammatory immune responses, including a higher Th17/rTreg cell ratio. These findings highlight the role of microbial metabolites in driving low-grade inflammation, which contributes to liver inflammation and fibrosis, positioning the gut-liver axis as a key factor in the progression of MASLD (43).

Similarly, Iino et al. (44) demonstrated that patients with MASLD exhibited diminished levels of *Faecalibacterium*, a beneficial bacteria known for producing SCFAs. This observation implies that a decrease in anti-inflammatory SCFAs such as butyrate could potentially facilitate the progression of the disease. The findings indicate that focusing on gut microbiota via dietary or probiotic interventions could serve as a viable management approach for MASLD (44). Astbury et al. provided insights into microbial diversity, revealing that patients with MASH, especially those with cirrhosis, showed a notable decrease in gut microbial diversity. The rise in *Collinsella* abundance was associated with higher triglycerides and cholesterol levels, whereas a reduction in Ruminococcaceae could potentially worsen the inflammatory conditions in MASH (45).

Boursier et al. further investigated the association between gut dysbiosis and M severity, revealing that the abundance of *Bacteroides* correlated with advanced fibrosis (F ≥ 2). At the same time, levels of *Prevotella* and *Ruminococcus* varied with disease stages. The observed microbial alterations were associated with metabolic pathways involving carbohydrates, lipids, and amino acids, indicating their possible role as biomarkers for disease severity and as targets for therapy (46).

Lanthier et al. conducted a study utilizing 16S rRNA sequencing, revealing a decreased abundance of *Clostridium* sensu stricto in patients with fibrosis, alongside an increase in Enterobacteriaceae and *Escherichia/Shigella*. The observed changes in the microbiome correlated with liver stiffness and muscle dysfunction, suggesting possible avenues for therapeutic intervention (47). Alferink et al. noted a decrease in microbial diversity among patients with MASLD who displayed steatosis, highlighting a relationship between specific bacterial genera, such as *Coprococcus* and *Ruminococcus gauvreaui*, and alterations in metabolic pathways, especially regarding bile acid synthesis and biotin metabolism. The results demonstrate that the variety and functionality of gut microbiota are essential in developing MASLD (48).

In a similar vein, Loomba et al. utilized whole-genome sequencing to identify microbial signatures that distinguish advanced fibrosis from less severe stages of MASLD, underscoring the potential of microbiome-based diagnostics for early disease detection (49). Oh et al. demonstrated the effectiveness of microbial and metabolomic signatures as diagnostic tools for cirrhosis, achieving high accuracy when integrated with clinical markers such as serum albumin and aspartate aminotransferase (AST) levels (50). Furthermore, Da Silva et al. emphasized changes in the gut microbiome, noting a decrease in *Ruminococcus* and *Faecalibacterium prausnitzii*, which occurred independently of obesity and insulin resistance. The observed changes were associated with modified metabolite profiles, including increased levels of propionate and 2-hydroxybutyrate, which further suggest a role for gut dysbiosis in the progression of MASLD and MASH (51).

Hoyles et al. established a connection between the enrichment of Proteobacteria and a decrease in microbial diversity, which is associated with hepatic inflammation and changes in lipid metabolism. Interventions like fecal microbiota transplants have shown promise in reducing steatosis and associated metabolic disturbances (52). Behary et al. investigated dysbiosis in MASLD-cirrhosis and its advancement to HCC, demonstrating that immune modulation induced by gut microbiota promotes T-cell suppression, which plays a role in disease progression (53). Caussy et al. identified a collection of microbial traits that distinguish MASLD-cirrhosis from non-cirrhosis, showcasing significant diagnostic precision and emphasizing the microbiome as a non-invasive biomarker for liver disease (54).

Ponziani et al. demonstrated a connection between gut dysbiosis and elevated inflammatory markers, alongside changes in bacterial composition, such as an increase in *Bacteroides* and a decrease in *Akkermansia*, in patients with MASLD-related HCC (55). Yang et al. noted a reduction of microbial diversity along with notable alterations in gut microbiota and metabolites, especially lipid molecules, in the context of metabolic-associated steatosis liver disease (MASLD). The results underscore possible pathways for diagnosis and treatment (56). León-Mimila et al. illustrated connections between metabolites derived from the microbiome, such as TMAO, and MASLD, especially among diabetic patients, highlighting the interaction of metabolic and microbiome elements in the advancement of the disease (57). Subsequent investigations, such as those conducted by Chen et al. and Nimer et al., explored bile acid metabolism in MASLD and MASH, revealing modified profiles and their links to fibrosis, inflammation, and genetic predispositions (58, 59). Lastly, Puri et al. and Lee et al. provided insights into the alterations in primary and secondary bile acids, alongside variations in microbial diversity associated with the severity of fibrosis, presenting further biomarkers for disease progression (60). The expanding collection of evidence underscores the complex interactions among the gut microbiome, its metabolites, and the development of MASLD/MASH, presenting new possibilities for diagnostics and treatment strategies.

**FIGURE 3:**
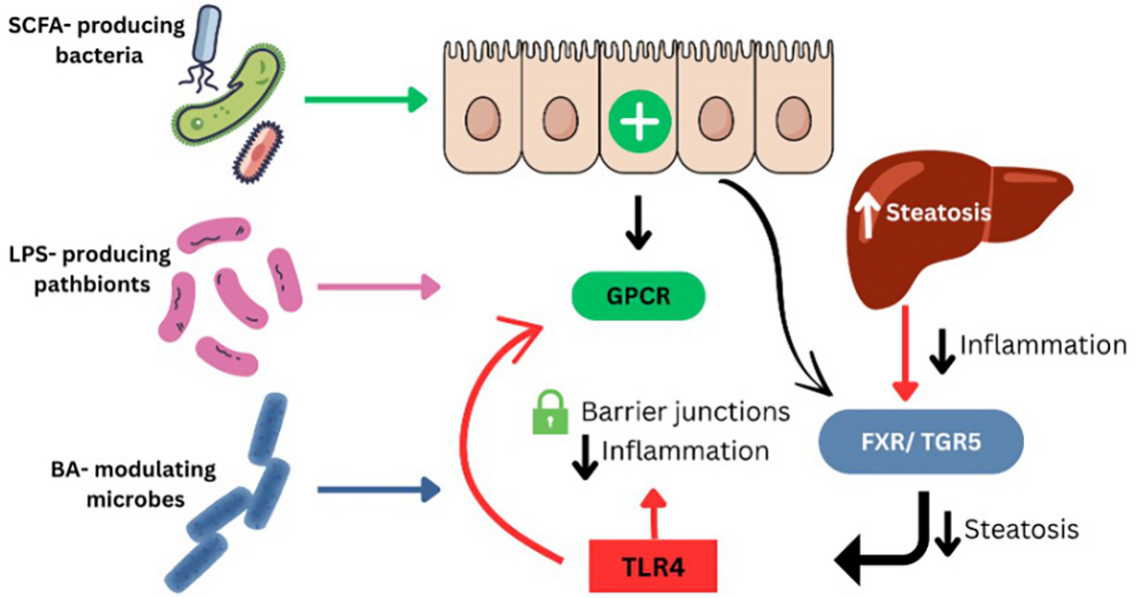
Gut microbiome-derived mechanisms contributing to MASLD pathogenesis. SCFA-producing bacteria enhance intestinal barrier integrity and reduce inflammation via activation of GPCR signaling. LPS-producing pathobionts increase gut permeability and trigger hepatic inflammation through TLR4. Bile acid–modulating microbes influence hepatic metabolism and inflammation through FXR and TGR5 signaling pathways. Disruption of these microbial functions promotes steatosis, immune dysregulation, and liver injury in MASLD.

Recent tissue-specific microbiome analyses have expanded our understanding of MASLD pathogenesis beyond stool-based data. In a 2024 metagenomic study of liver and adipose samples from MASLD patients, taxa such as *Enterococcus*, *Granulicatella*, and members of the Morganellaceae family were enriched in hepatic tissue and correlated with pro-inflammatory gene signatures, histological steatohepatitis, and fibrosis severity (61). These findings support a growing model where tissue-resident microbiota may contribute directly to liver inflammation and immune modulation in MASLD (Figure 3). In a recent large-scale meta-analysis, Nychas et al. (62) identified a robust set of gut microbial signatures for NAFLD across multiple international cohorts using harmonized metagenomic datasets. Taxa such as *Acinetobacter*, *Faecalibacterium*, and *Enterococcus* were consistently associated with NAFLD severity and fibrosis progression. These reproducible microbial markers support the clinical utility of gut microbiome profiling for MASLD stratification and diagnosis (62).

#### Microbiome-derived metabolites and host pathophysiology

Microbial metabolites such as SCFAs and bile acids play key roles in host metabolism, gut barrier integrity, and hepatic inflammation (Figure 3). Because these mechanisms are more thoroughly characterized in human studies, we refer the reader to the Section “Human Studies” for a detailed explanation of their roles in MASLD and MASH (Table 2).

**TABLE 2:** Role of short-chain fatty acids (SCFAs) and other gut microbial metabolites in MASLD/MASH pathogenesis.

SCFAs	Effect on MASLD/MASH	Mechanism	Reference
Acetate	Enhances gut barrier integrity, reduces liver inflammation and fibrosis	Promotes mucin production, strengthens gut barrier, prevents translocation of harmful bacteria and endotoxins	(118)
Propionate	Inhibits cholesterol synthesis, promotes lipid oxidation, reduces hepatic fat accumulation	Activates G-protein-coupled receptors (GPR41 and GPR43), inhibits histone deacetylases (HDACs)	(119)
Butyrate	Strengthens gut barrier, reduces inflammation, regulates lipid metabolism	Enhances mucosal barrier function by increasing mucin and IgA production, facilitates regulatory T cell differentiation by inhibiting HDACs, prevents intestinal inflammation	(120)
**Other metabolites**			
Trimethylamine N-oxide (TMAO)	Promotes atherosclerosis and liver inflammation, linked to increased risk of cardiovascular diseases and liver disease progression	Produced from dietary choline and carnitine by gut bacteria	(121)
Secondary bile acids	Dysregulation can exacerbate liver injury affects lipid and glucose metabolism by inhibiting FXR signaling.	Produced by gut microbiota from primary bile acids	(122)
Indoles	They have anti-inflammatory and antioxidant properties, modulate gut barrier function and immune responses	Derived from the metabolism of tryptophan by gut bacteria	(123)

This table summarizes the metabolic pathways and functional roles of key gut-derived compounds, including SCFAs, TMAO, ethanol, and bile acids, and their association with disease mechanisms such as inflammation, lipid accumulation, and fibrosis.

The majority of human studies included in this review were cross-sectional, limiting the ability to assess temporal shifts in gut microbial composition or determine causality. While these studies provide valuable associations between microbiome profiles and MASLD/MASH severity, they do not allow for longitudinal tracking of microbiome changes during disease progression or treatment response. This reflects a methodological gap that constrains interpretation of dynamic microbiome–host interactions in MASLD.

A significant limitation in the current MASLD microbiome literature is the lack of longitudinal human studies. Most data are derived from cross-sectional cohorts, which provide only a snapshot in time and cannot establish whether gut microbiome alterations are causes, consequences, or correlates of disease. This design limitation hampers our understanding of how microbial patterns evolve from early steatosis to advanced fibrosis or MASH, and how they respond to interventions such as dietary modification, weight loss, or probiotic supplementation. Without prospective follow-up, it is difficult to identify predictive microbial markers or determine the durability of microbiome modulation. Future research should prioritize longitudinal and interventional studies to address these gaps and enable microbiome-based stratification and therapy in MASLD.

## Discussion

### Innovative techniques in gut microbiome analysis

Multiple complementary approaches have been developed to study the gut microbiome in MASLD/MASH, ranging from culture-independent high-throughput sequencing to culture-based innovations. These include targeted sequencing (e.g., 16S rRNA, 18S rRNA, ITS), untargeted methods such as shotgun metagenomics and metagenomic next-generation sequencing (mNGS), and complementary omics such as transcriptomics, proteomics, metabolomics, and culturomics. Each technique varies in resolution, functional insights, and limitations, and can be combined to generate a holistic understanding of microbial communities and their interactions with the host. Table 3 summarizes the key methodologies, their primary applications, advantages, and limitations in the context of MASLD research.

**TABLE 3:** Innovative techniques for gut microbiome analysis in MASLD/MASH research, including their primary applications, key methdological features and limitations.

Technique	Purpose/application	Key features	Limitations	References
16S rRNA amplicon sequencing	Bacterial taxonomic profiling	Cost-effective; detects unculturable species	Limited strain-level resolution; bacteria/archaea only	(48, 131)
18S rRNA/ITS sequencing	Fungal profiling	Targets eukaryotic microbes	Limited databases; amplification bias	(132, 133)
Shotgun metagenomics (mNGS)	Comprehensive taxonomic + functional profiling	Detects bacteria, viruses, fungi, genes/pathways	Expensive; requires high computing power	(132, 133)
Whole genome sequencing	Strain-level genome characterization	High-resolution functional data	Requires culture; labor-intensive	(132, 133)
Metatranscriptomics	Active gene expression profiling	Captures real-time microbial activity	RNA instability; high cost	(132, 133)
Proteomics	Microbial protein identification	Functional insight into expressed proteins	Technically demanding; may miss low-abundance proteins	(134)
Metabolomics (targeted/untargeted)	Profiling metabolites (e.g., SCFAs, bile acids)	Reflects host–microbiome interactions	Sensitive to sample handling; complex analytics	(134)
Culturomics	Isolation and characterization of microbes	Expands cultured species diversity	Time-consuming; biased toward culturable taxa	(135, 136)
Biomarker discovery frameworks	Identify taxa/pathways linked to disease	Statistical and machine learning approaches	Requires robust, phenotyped cohorts	(137)
Multi-omics integration	Combine microbiome with other omics	Tools like MixOmics, MOFA+, DIABLO	Computationally complex; high data demands	(48, 56, 138)

### Microbial taxa correlated with MASLD and MASH: a tabular analysis

The intricate relationship between the gut microbiome and the liver in MASLD and MASH encompasses several microbial taxa and their corresponding metabolites. Recent advancements in sequencing technologies have enabled more thorough investigations of the gut metagenome, yielding fresh insights into the specific microbial species and roles implicated in liver disorders (63). One of the most regularly observed modifications in the gut microbiome occurs in patients with MASLD and MASH. A substantial alteration is the elevated ratio of Firmicutes to Bacteroidetes, associated with improved energy extraction from food and changes in bile acid metabolism, both of which are contributing factors to the development of fatty liver disease (64, 65).

### Disease stage-specific microbiome signatures

Certain genera within the Firmicutes phylum have been linked to MASLD and MASH. *Ruminococcus* is frequently present in greater quantities in MASLD patients, corresponding with increased fibrosis severity. *Ruminococcus* species generate acetate, a substrate that can enhance hepatic lipogenesis, consequently promoting fat storage in the liver (34, 37). Certain genera within the Firmicutes phylum have been linked to MASLD and MASH. *Ruminococcus* is frequently present in greater quantities in MASLD patients, corresponding with increased fibrosis severity. *Ruminococcus* species generate acetate, a substrate that can enhance hepatic lipogenesis, consequently promoting fat storage in the liver (34, 37). In contrast, specific advantageous bacterial taxa are reduced in patients with MASLD and MASH.

The role of *Faecalibacterium prausnitzii* in MASLD and MASH–particularly its depletion and involvement in SCFA-mediated anti-inflammatory pathways–is summarized in Table 4. The Proteobacteria phylum, particularly the Enterobacteriaceae family, has been identified as enriched in individuals with MASLD and MASH. These gram-negative bacteria are distinguished by their synthesis of LPS, which can trigger inflammatory responses that worsen liver disease. An elevated presence of *Escherichia coli*, a constituent of the Enterobacteriaceae family, is associated with higher liver inflammation and fibrosis severity in patients with MASH (66, 67). This indicates that dysbiosis within this bacterial group may be crucial in the pathogenesis of liver disorders. A notable group is the Bacteroidetes phylum, especially the genus *Bacteroides*. Although the total quantity of Bacteroidetes is frequently diminished in MASLD patients, specific species, such as *Bacteroides vulgatus*, have shown a favorable correlation with the severity of MASLD. This link may arise from its activity fostering intestinal inflammation and impairing barrier integrity, potentially resulting in heightened translocation of bacteria and their byproducts into the bloodstream, exacerbating hepatic inflammation (68, 69). Interstingly, while several studies indicate a reduction in Bacteroidetes among MASLD patients, others suggest that particular species may proliferate as the severity of the disease intensifies (36). Conversely, individuals with MASLD and MASH typically exhibit a reduction in members of the Actinobacteria phylum, especially the species *Bifidobacterium*. *Bifidobacterium* species are often seen as advantageous due to their capacity to generate short-chain fatty acids such as acetate and lactate, which can then be transformed into butyrate by other intestinal bacteria. Butyrate is recognized for its preventive properties regarding intestinal health and may assist in alleviating the progression of liver disease (67, 69). Particular microbial taxa have been associated with specific phases of MASLD development. *Lactobacillus* species are typically more prevalent in patients with mild steatosis, but genera like Oscillospira and Dorea are enriched in individuals with MASH. This indicates that distinct bacterial populations may assume specific roles during the different stages of liver disease (43, 70). The association between gut microbial taxa and MASLD and MASH is intricate and encompasses numerous metabolites that affect liver function and disease advancement. Specific bacteria in the gut microbiota can generate ethanol via fermentation, leading to oxidative stress and hepatic damage. The presence of *Desulfovibrio piger*, a hydrogen sulfide-producing species, is higher in MASH patients, potentially worsening intestinal inflammation and impairing intestinal barrier function (71–73).

**TABLE 4:** Microbial taxa associated with MASLD and MASH: correlations, proposed mechanisms, and functional roles.

Microbial taxa	Association with MASLD/MASH	Mechanism of action/association	References
Firmicutes (phylum)	Increased in MASLD/MASH.	Increased energy extraction from the diet and modified bile acid metabolism facilitates fat storage in the liver, advancing the course of MASLD.	(124, 125)
Bacteroidetes (phylum)	Decreased in MASLD/MASH.	Decreased synthesis of advantageous metabolites, including short-chain fatty acids (SCFAs), resulting in modified energy metabolism and heightened inflammation.	(37, 125)
*Ruminococcus* (genus)	Increased levels of MASLD correlated with advanced fibrosis.	Generates acetate, which acts as a substrate for hepatic lipogenesis, therefore intensifying liver fat formation.	(51, 126)
*Faecalibacterium prausnitzii*	Decresaed in MASLD.	Decreased butyrate synthesis attenuates its anti-inflammatory properties, exacerbating liver inflammation.	(16, 125)
Proteobacteria (phylum)	Increased in MASLD/MASH.	Primary source of LPS, which induce systemic inflammation and disease progression.	(37, 124)
*Escherichia coli*	Increased levels in MASH are associated with significant inflammation and fibrosis.	Generates LPS that exacerbates intestinal inflammation and impairs gut barrier integrity.	(125, 126)
*Bacteroides vulgatus*	Exhibits a positive correlation with the severity of MASLD.	Facilitates intestinal inflammation and compromises barrier integrity, leading to metabolic abnormalities.	(124)
*Bifidobacterium* (genus)	Decreased in MASLD/MASH.	Decreased synthesis of acetate and lactate results in impaired gut health and metabolic control.	(37, 125)
*Lactobacillus* (genus)	Increasing with uncomplicated steatosis.	May contribute to the initial phases of MASLD progression by affecting gastrointestinal health and metabolic functions.	(124, 126)
*Oscillospira* (genus)	Increased in MASH.	Linked to the advancement of more severe liver disease via the regulation of inflammatory pathways.	(37, 124)
*Dorea* (genus)	Increased in MASH.	Associated with advanced stages of MASLD, possibly via its impact on intestinal permeability and inflammation.	(124, 126)
*Desulfovibrio piger*	Increased in MASH.	Generates hydrogen sulfide, which exacerbates intestinal inflammation and may aggravate liver damage.	(124, 125)
*Clostridium* (genus)	Modified prevalence in MASLD/MASH.	Deconjugates bile acids, impacting lipid and glucose metabolism, hence affecting hepatic fat storage.	(37, 125)
Enterobacteriaceae (family)	Increased in MASLD/MASH.	Generates LPS and trimethylamine (TMA), which contribute to inflammation and metabolic disorders linked to liver disease.	(124)
Erysipelotrichaceae (family)	Increased in MASLD.	The synthesis of TMA may facilitate the development of TMAO, which is associated with cardiovascular risk factors that worsen liver disease.	(125, 126)

This table presents key gut microbial genera and species linked to MASLD and MASH, highlighting their direction of association (enriched or depleted), proposed mechanisms of action (e.g., SCFA production, endotoxin release), and potential clinical implications.

The intricate relationships among specific microbial taxa, their metabolites, and host factors play a significant role in the pathophysiology of liver disorders (16, 38). Comprehending the intricate connections between the gut microbiota and the pathogenesis of MASLD/MASH presents prospects for the development of innovative therapeutic strategies aimed at the gut microbiome for the prevention and treatment of these hepatic disorders (64, 74). Potential solutions encompass the utilization of probiotics, prebiotics, and other microbiome-modulating therapies to reestablish a healthy gut microbial equilibrium and enhance liver health (16, 74).

### Probiotics, prebiotics, and synbiotics in MASLD/MASH management: insights from current studies

Probiotics enhance gut microbiota composition, reduce inflammatory cytokines, and stabilize mucosal immune function in MASLD patients. The therapeutic effects of these interventions are partly mediated through SCFA production, gut barrier support, and inflammation regulation, as detailed in the Section “Human Studies” and Table 5.

**TABLE 5:** Effects and mechanisms of probiotics, prebiotics, and synbiotics in MASLD and MASH.

Biotic type	Effect	Mechanism of action	Citation
Probiotic	Enhances gut microbiota composition, reduces inflammatory cytokines, and stabilizes mucosal immune function in MASLD patients.	Modulates gut microbiota composition to improve microbial diversity decreases inflammatory cytokines like IFN-γ and TNF-α and stabilizes mucosal immune function.	(127)
	Modulates the gut microbiota, thereby supporting intestinal and extra-intestinal health and mitigating microbial	Enhances the growth of beneficial microbes and reduces harmful bacteria to restore intestinal microbial homeostasis.	(128)
	Establishes guidelines for the precise definition and clinical application of probiotics to maximize their therapeutic potential.	Advocates the standard use of probiotics to target microbial dysbiosis and improve host health through gut microbiota modulation.	(129)
	Improves gut microbiota composition and reduces hepatic fat accumulation and inflammatory markers, offering a promising strategy for MASLD management.	Promotes beneficial gut bacteria, decreases gut permeability, reduces endotoxin production, and modulates inflammatory pathways via the gut-liver axis.	(75)
	Demonstrates a link between gut microbiome-derived metabolites and genetic effects associated with hepatic steatosis and fibrosis in MASLD.	Explores interactions between gut-derived metabolites and host genetics to impact liver steatosis and fibrosis progression.	(76)
Prebiotic	Improves metabolic health by enhancing gut microbiota composition, including increased beneficial bacteria, and modulating host energy homeostasis.	Selectively stimulates beneficial bacteria like *Bifidobacteria* and *Lactobacillus*, reduces dysbiosis, and impacts glucose and lipid metabolism.	(77)
	Promotes the growth of colonic butyrate-producing bacteria and bifidobacteria, influencing metabolic pathways linked to gut and systemic health.	Supports the growth of butyrate-producing bacteria, enhancing energy homeostasis and modulating gut environment	(78)
	Selectively stimulates beneficial gut bacteria, improving host well-being and gastrointestinal health by optimizing bacterial activity.	Enhances the activity of gut bacteria, specifically *Bifidobacteria*, to promote improved gut microbial balance and activity.	(79)
	Facilitates the metabolic conversion of prebiotics by gut bacteria into beneficial metabolites, such as organic acids, thereby enhancing gut health.	Prebiotics are metabolized by gut bacteria like *Bifidobacteria* and *Lactobacillus* to produce organic acids and SCFAs, influencing gut health and fermentation pathways.	(80)
	Strengthens the intestinal barrier by promoting tight junction assembly through AMP-activated protein kinase activation.	Activates AMPK signaling pathways to facilitate tight junction protein assembly, enhancing intestinal barrier integrity.	(81)
Synbiotic	Significantly reduces liver enzymes, improves lipid profiles, decreases obesity indices, and lowers inflammatory markers in MASLD patients.	Combines probiotics and prebiotics to enhance the gut-liver axis, improving hepatic metabolism, lipid profiles, and systemic inflammation.	(82)
	Defines and expands the scope of synbiotics as mixtures of probiotics and prebiotics with complementary or synergistic effects to enhance gut health and confer host benefits.	Combines complementary or synergistic actions of probiotics and prebiotics, enhancing the selective utilization of substrates by gut microbes for host benefit.	(130)

This table summarizes microbiome-targeted interventions and their roles in modulating gut composition, reducing inflammation, enhancing barrier integrity, and improving liver-related outcomes.

Prebiotics improve metabolic health by enhancing the growth of beneficial microbes such as *Bifidobacterium* and *Lactobacillus*. Their benefits are similarly linked to SCFA-mediated effects and improved microbial balance, which support liver health as summarized earlier.

Synbiotics combine probiotics and prebiotics to synergistically modulate the gut microbiome. These synergistic effects contribute to gut–liver axis improvement and metabolic regulation, supported by the microbial mechanisms detailed in Table 5.

### Fecal microbiota transplantation (FMT) for MASLD and MASH: assessing its therapeutic potential and constraints

Fecal microbiota transplantation (FMT) has emerged as a novel therapeutic approach for gastrointestinal and metabolic disorders, including MASLD and MASH. FMT involves the transfer of fecal material from a healthy donor to a recipient, intending to restore a balanced gut microbiome (75). This procedure has gained attention due to its potential to improve metabolic health by addressing dysbiosis–a common feature in individuals with MASLD and MASH. Numerous studies have demonstrated the efficacy of FMT in patients with liver diseases, particularly in restoring gut microbiota diversity and improving liver function. A groundbreaking study conducted in Saudi Arabia highlighted the potential benefits of FMT for patients with MASH (76). In this clinical trial, patients who received FMT exhibited significantly improved liver function tests, metabolic profiles, and overall gut health compared to the control group. Similar findings have been observed globally. For instance, a systematic review by Gu X et al. (77) analyzed multiple clinical trials and reported that FMT resulted in significant reductions in liver enzymes, including alanine aminotransferase (ALT) and aspartate aminotransferase (AST), indicating improved liver function. Furthermore, improvements in insulin sensitivity and reductions in body weight were also noted, suggesting that FMT may exert systemic metabolic benefits.

The mechanisms through which FMT exerts its therapeutic effects on liver diseases are multifaceted. One primary mechanism is the restoration of microbial diversity. Individuals with MASLD often exhibit reduced microbial diversity and altered gut microbiota composition, characterized by an overrepresentation of pathogenic bacteria and a deficiency of beneficial species (78). FMT has been shown to restore this diversity, leading to the re-establishment of a healthy gut microbiome. Moreover, FMT enhances gut barrier function, critical in preventing the translocation of harmful bacteria and their byproducts into the bloodstream. Dysbiosis can lead to increased intestinal permeability, allowing bacterial endotoxins, such as LPS, to enter circulation and trigger systemic inflammation (79, 80). By restoring healthy microbiota, FMT can improve gut barrier integrity, reducing the risk of inflammation and liver injury. Additionally, FMT has been shown to modulate immune responses in the gut and liver. A study by (81) reported that FMT could significantly reduce inflammatory markers in patients with MASH. This immunomodulatory effect is crucial in mitigating the chronic inflammation associated with MASLD and MASH, ultimately leading to improved liver health.

Despite the promising results associated with FMT, several challenges and limitations must be considered. One significant concern is the variability in donor microbiota. The composition of the donor microbiome can significantly influence the efficacy of FMT. For example, differences in dietary habits, lifestyle factors, and genetics can lead to variations in microbial composition between donors, potentially affecting treatment outcomes (82). Moreover, the risk of transmission of infectious agents during FMT is a critical concern. While rigorous screening protocols for donors are implemented to minimize this risk, there remains a potential for transmission of pathogens that may not be detected during screening (83). Reports of adverse events following FMT, such as infections and gastrointestinal complications, emphasize the need for careful donor selection and monitoring of recipients. Another limitation of FMT is the lack of standardization in protocols. Variations in the fecal processing, preparation, and administration method can lead to inconsistent results across studies. A consensus on best practices for FMT is essential for establishing its clinical efficacy and safety (84). Despite these challenges, ongoing research continues to explore the potential of FMT in managing MASLD and MASH. Future studies should focus on identifying optimal donor characteristics and developing standardized protocols to enhance the safety and efficacy of FMT. Additionally, investigating the long-term effects of FMT on liver health and metabolic outcomes is crucial to understanding its role in managing MASLD/MASH.

Furthermore, the exploration of alternative microbiome-based therapies, such as targeted probiotics or microbial consortia, may provide safer and more controlled options for modulating the gut microbiome without the risks associated with FMT. A recent study by (85) demonstrated the potential of specific probiotic formulations to mimic the beneficial effects of FMT, suggesting that these therapies may serve as viable alternatives.

The application of FMT in managing MASLD and MASH is not confined to any specific region. Studies from Asia, Europe, and North America have explored its efficacy, highlighting the importance of diverse microbiomes across populations. For example, a study in Japan demonstrated significant improvements in liver function and metabolic parameters in patients with MASH following FMT (64).

In conclusion, FMT represents a promising therapeutic strategy for managing MASLD and MASH, with evidence supporting its efficacy in improving liver function and metabolic health. However, challenges related to donor variability, safety concerns, and the need for standardized protocols must be addressed. Ongoing research and clinical trials will be essential in uncovering the full potential of FMT and alternative microbiome-based therapies in managing these increasingly prevalent liver diseases.

### Dietary interventions and gut microbiome dynamics: implications for MASLD and MASH therapy

Dietary interventions play a pivotal role in managing MASLD and MASH as they significantly influence the composition and function of the gut microbiome. The gut microbiota, comprising trillions of microorganisms, is crucial for maintaining metabolic health and homeostasis. Dietary patterns, particularly those rich in fiber, healthy fats, and low in refined sugars, can profoundly affect gut microbial diversity and functionality, leading to improved liver health outcomes (86). Research has shown that the gut microbiome can influence liver health through several mechanisms. These effects are partly mediated through microbial metabolites such as SCFAs, which are discussed in Table 2.

Recent studies have further emphasized the complex relationship between dietary factors and the gut microbiome in the development and management of MASLD and MASH. Dietary patterns rich in fiber and polyphenols have been shown to enhance the growth of beneficial microbial taxa such as *Akkermansia muciniphila* and *Faecalibacterium prausnitzii*, which are inversely associated with hepatic fat accumulation and systemic inflammation (36). These dietary-induced microbial shifts lead to increased production of SCFAs and improved gut barrier integrity, thereby reducing endotoxemia and liver inflammation. Adherence to a green-Mediterranean diet, characterized by high intake of plant-based foods and limited animal proteins, has been linked to favorable changes in gut microbiota composition and significant reductions in intrahepatic fat, independent of weight loss (87). Furthermore, gut dysbiosis has been implicated in the pathogenesis of MASLD/MASH through mechanisms involving increased intestinal permeability and translocation of microbial-derived endotoxins, which activate inflammatory pathways in the liver (37). These findings highlight the crucial role of the gut-liver axis and underscore the importance of dietary strategies that restore microbial balance to support liver health in MASLD/MASH patients.

Emerging therapeutic strategies for metabolic dysfunction-associated steatotic liver disease (MASLD) and metabolic dysfunction-associated steatohepatitis (MASH) increasingly center on incretin-based agents, particularly dual glucose-dependent insulinotropic polypeptide (GIP) and glucagon-like peptide-1 (GLP-1) receptor agonists, such as tirzepatide, and GLP-1/glucagon receptor co-agonists like survodutide. These agents have garnered attention due to their potential to target the metabolic dysfunctions that underpin the pathogenesis of MASLD/MASH (88).

Tirzepatide, a novel dual GIP and GLP-1 receptor agonist, has shown promising efficacy in modulating biomarkers associated with non-alcoholic steatohepatitis (NASH) among individuals with type 2 diabetes. In a 26-week randomized, placebo-controlled trial, tirzepatide significantly reduced serum levels of alanine aminotransferase (ALT), aspartate aminotransferase (AST), cytokeratin-18 (K-18), and procollagen type III (Pro-C3), while concurrently increasing levels of adiponectin, a marker of insulin sensitivity and anti-inflammatory activity. The reductions in K-18 and Pro-C3 were both dose-dependent and statistically significant, suggesting that tirzepatide may attenuate hepatic injury and fibrogenesis. These findings support the therapeutic potential of tirzepatide in the management of MASLD/MASH through metabolic and anti-inflammatory mechanisms (88).

Survodutide, a novel GLP-1/glucagon receptor co-agonist, is currently undergoing clinical evaluation for its therapeutic potential in metabolic dysfunction-associated steatohepatitis (MASH). Interim results from a phase 2 randomized clinical trial have demonstrated that survodutide significantly reduces hepatic steatosis, body weight, and markers of hepatic fibrosis, while also improving metabolic parameters. The agent exhibited a safety profile comparable to other incretin-based therapies, with good overall tolerability. These preliminary findings underscore survodutide’s potential as a promising pharmacologic candidate for the treatment of MASH, particularly in individuals with concomitant obesity or features of metabolic syndrome, where metabolic modulation plays a central role in disease progression (89).

Increasing dietary fiber intake is one of the most effective strategies for improving gut health and managing MASLD/MASH. Dietary fibers are fermented in the colon, leading to the production of SCFAs, which play a crucial role in modulating inflammation and improving gut barrier function (90). A recent study found that higher fiber intake was associated with increased fecal SCFA concentrations and improved liver health in patients with MASLD (91). The intake of omega-3 and omega-6 fatty acids has been shown to impact liver health positively. Omega-3 fatty acids, found in fatty fish and certain plant oils, possess anti-inflammatory properties and have been associated with improved liver function (92). Research indicates that increasing omega-3 intake can reduce liver fat and improve insulin sensitivity in individuals with MASLD (93). A study conducted in Saudi population demonstrated that participants who consumed more omega-3-rich foods showed significant improvements in liver enzyme levels and reduced hepatic fat (94).

A systematic review highlighted that increasing antioxidant-rich foods in the diet is associated with improved liver health and reduced inflammation in MASLD patients (95). This effect may be particularly important in the context of populations in Asia and the Gulf region, where dietary habits can be tailored to include more antioxidant-rich foods. Personalized nutrition is an emerging concept that recognizes the unique dietary needs of individuals based on their genetic, environmental, and microbiome profiles. Research suggests that tailored dietary interventions can significantly enhance the management of MASLD/MASH by considering individual differences in gut microbiota composition and metabolic responses (17). A study conducted in the Gulf region demonstrated that personalized dietary recommendations based on microbiome analysis resulted in improved metabolic outcomes and liver health in patients with MASLD (96). This approach holds great promise for optimizing dietary interventions and enhancing patient adherence to dietary recommendations.

### Environmental factors, gut microbiome, dietary influences, and lifestyle impacts on MASLD in the MENA region

The Middle East and North Africa (MENA) region has rapidly urbanized in recent decades, triggering significant shifts toward sedentary lifestyles, increased obesity, and higher type 2 diabetes (T2D) prevalence. The MENA region’s T2D rate of approximately 12.3% surpasses the global average of 9.3%, and predictions estimate a further increase of 25% by 2030 and 51% by 2045 due to continued urbanization and dietary shifts (97). Urban populations in Saudi Arabia, for example, show T2D prevalence rates as high as 32%, reflecting the direct impact of urban lifestyles on metabolic health (98). Obesity prevalence in Saudi adults has notably reached around 41%, directly correlating with MASLD risk, making the condition highly prevalent, estimated to affect approximately 44% of adults over 20 years (97) (Table 6).

**TABLE 6:** Comparison of epidemiological and lifestyle factors between the MENA region and Saudi Arabia.

Aspect	MENA (general)	Saudi Arabia (specific)	Key differences	References
Urbanization rate	High urbanization, with variation across countries	Extremely high urbanization, accompanied by rapid industrialization	Saudi Arabia demonstrates higher urbanization intensity and industrial development	(97)
Dietary patterns	Transitional diets – mix of traditional and modern elements	Strong shift toward Western dietary patterns, including high consumption of processed foods	Saudi Arabia exhibits a more rapid and pronounced Westernization of diet	(98)
Physical activity levels	Generally low; varies based on environmental and cultural context	Significantly lower, driven by climatic factors and sedentary lifestyle	Saudi Arabia reports markedly lower physical activity levels	(98)
Prevalence of obesity	High – ∼60% overweight or obese regionally	Very high – ∼41% obesity rate among adults	Obesity prevalence is especially elevated in Saudi Arabia	(97)
Prevalence of NAFLD/NASH	Varies between 30% and 40%	Exceptionally high – ∼44% in adults, with rapidly increasing trends	Saudi Arabia faces one of the highest NAFLD/NASH burdens globally	(97)
Major environmental pollutants	Common: air pollution, endocrine-disrupting chemicals (EDCs), heavy metals, and pesticides	Elevated urban air pollution and significant exposure to EDCs	Saudi Arabia experiences more concentrated exposure to air pollutants and endocrine disruptors	(139)
Cultural and dietary practices	Diverse; includes late meals influenced by cultural heterogeneity	Regular late-night eating; Ramadan fasting shown to significantly impact metabolism and liver function	Ramadan and cultural practices play a distinct role in shaping metabolic responses in Saudi Arabia	(107)
Public health interventions	Variable and inconsistently implemented	Expanding targeted initiatives focused on obesity, diabetes, and metabolic syndrome prevention	More structured and aggressive public health approaches are being deployed in Saudi Arabia	(97, 98)

This table highlights key differences in obesity prevalence, dietary patterns, physical activity levels, and metabolic risk factors that may influence gut microbiome composition and MASLD/MASH development.

### Dietary patterns and MASLD

Dietary transitions from traditional nutrient-rich diets toward calorie-dense Western-style diets have significantly influenced metabolic dysfunction and liver health in MENA. Western dietary patterns characterized by high intake of refined sugars, saturated fats, and processed foods have consistently been linked to a 56% increased risk of NAFLD (99). Fructose-rich diets, prevalent in soft drinks and sweetened beverages, particularly exacerbate hepatic steatosis by enhancing lipogenesis and insulin resistance, thus intensifying MASLD severity (99). The Mediterranean diet (MedDiet), characterized by high consumption of fruits, vegetables, whole grains, legumes, nuts, and olive oil, has been linked to numerous health benefits, including improved liver function. Studies indicate that adherence to this diet is associated with lower rates of MASLD and better metabolic profiles. Low-carbohydrate diets, particularly those that restrict refined sugars and grains, have gained popularity in recent years for their potential benefits in managing MASLD/MASH. Research indicates that reducing carbohydrate intake can significantly improve liver fat content and overall metabolic health (100). A study in the Gulf region demonstrated that a low-carbohydrate, high-fat diet resulted in improved liver function markers and reduced hepatic steatosis in patients with (101). Therefore, adherence to the (MedDiet), rich in fiber, polyphenols, and unsaturated fats, significantly improves liver function and reduces steatosis and inflammation in MASLD patients (102). Polyphenols such as resveratrol and curcumin modulate the gut microbiota positively, reducing hepatic inflammation and oxidative stress (102). A meta-analysis highlighted that MedDiet adherence correlates with improved hepatic biomarkers, reduced fibrosis, and lower NAFLD prevalence (103, 104).

Different types of dietary fiber have distinct fermentation profiles that influence SCFA production and thereby modulate MASLD progression. Inulin-type fructans tend to promote acetate and butyrate, whereas resistant starch more selectively enhances butyrate levels. Acetate, while beneficial in moderation, can serve as a substrate for hepatic lipogenesis, potentially exacerbating steatosis, whereas butyrate supports gut barrier function and reduces hepatic inflammation. Therefore, fibers that favor butyrate production–like resistant starch–may offer superior therapeutic benefit in MASLD by enhancing gut–liver axis integrity without promoting lipogenesis. These fermentation-specific effects should be considered in future nutritional intervention strategies (105).

### Lifestyle and cultural factors

Physical inactivity, prevalent throughout the MENA region, strongly influences MASLD progression. More than 40% of adults in Arab countries fail to achieve recommended physical activity levels, exacerbating insulin resistance and liver fat accumulation (106). Conversely, moderate exercise consistently demonstrates significant improvements in hepatic steatosis, inflammation, and insulin sensitivity, underscoring the critical role of physical activity in MASLD management (106).

Cultural meal timing, particularly night-time heavy meals and irregular eating patterns, negatively impacts hepatic metabolism and MASLD outcomes. In contrast, intermittent fasting practices, such as Ramadan fasting, positively influence metabolic parameters, weight reduction, and hepatic health, suggesting beneficial circadian rhythm adjustments in MASLD patients (107).

### Environmental chemical exposure

Environmental contaminants significantly contribute to MASLD by altering gut microbiota and hepatic metabolism. Chronic exposure to air pollution (PM2.5, NOx) correlates strongly with increased NAFLD prevalence and severity, mediated through systemic inflammation, gut barrier disruption, and endotoxemia (108). Additionally, persistent organic pollutants (POPs) and endocrine-disrupting chemicals (EDCs), such as pesticides, PCBs, BPA, and phthalates, directly exacerbate MASLD via gut microbiota dysbiosis, increased intestinal permeability, oxidative stress, and hepatic inflammation (109). Animal models consistently demonstrate worsened steatosis, insulin resistance, and hepatic inflammation following chronic EDC exposure, indicating significant environmental contribution to MASLD progression (109).

In Saudi Arabia, traditional diets are often high in carbohydrates and saturated fats, contributing to the rising prevalence of MASLD. However, recent shifts toward healthier dietary patterns, such as the Mediterranean diet, have shown promising results in managing liver health (101). Research indicates that adopting such diets can significantly improve liver function and metabolic parameters. Asian countries, particularly East Asian ones, have distinct dietary patterns that influence liver health. The traditional Asian diet, rich in rice, vegetables, and fish, may provide protective effects against MASLD due to its high fiber content and beneficial fatty acid profile (110). Studies from Japan have shown that adherence to traditional dietary practices is associated with lower rates of MASLD and better metabolic health outcomes. Globally, dietary interventions are being recognized as key components in the management of MASLD/MASH. The evidence supports that diets low in saturated fats and high in fruits, vegetables, and whole grains improve liver health and metabolic outcomes. Collaborative efforts across countries can enhance the understanding of dietary influences on liver diseases and facilitate the sharing of effective dietary strategies.

Overall, dietary interventions play a crucial role in modulating the gut microbiome and improving outcomes for individuals with MASLD and MASH. Patients can significantly enhance their liver health and overall well-being by adopting diets rich in fiber, healthy fats, and antioxidants. Personalized dietary strategies based on individual microbiome profiles hold great promise for optimizing treatment outcomes. Ongoing research and collaboration among countries will be essential to further elucidate the relationship between diet, gut microbiota, and liver health.

### Regional comparisons and the MENA gap

To our knowledge, this is the first systematic review to synthesize both human and animal MASLD/MASH microbiome data with explicit attention to underrepresented MENA-region populations. Although global studies of MASLD/MASH consistently show reduced microbial diversity, decreased abundance of SCFA producers (e.g., *Faecalibacterium*, *Roseburia*), and enrichment of pathobionts like *Escherichia*, evidence from the MENA region remains sparse. The only identified study from the Arabian Peninsula focused on Arab Kuwaitis, finding Firmicutes and Bacteroidetes as dominant phyla (111). Moreover, recent efforts to expand human gut microbiome references in underrepresented populations (e.g., India, Japan, Korea) reveal significant geographic bias in existing catalogs. Cultural, dietary, and genetic factors–such as limited alcohol consumption, high refined-carbohydrate diets, and consanguinity–may shape region-specific microbiome patterns but are currently underexplored in MASLD research. These gaps highlight the urgent need for high-quality, population-specific studies in Arab and Middle Eastern populations.

## Limitations

This review is subject to several limitations. First, the heterogeneity in study designs, inclusion criteria, patient demographics, sample sizes, sequencing platforms, and microbiome analysis methods (e.g., 16S vs. shotgun metagenomics) limits direct comparability across studies. Second, we included only English-language publications, which may have excluded relevant findings reported in other languages. Third, although this review aimed to assess both global and MENA-specific studies, there was a disproportionate representation from non-MENA regions, limiting the ability to perform a robust geographic stratification. The regional microbiome findings from the MENA cohort remain underpowered and should be interpreted with caution. Fourth, this review is based on a narrative synthesis rather than a quantitative meta-analysis, due to methodological variability and inconsistent outcome reporting across studies. Fifth, there was a lack of longitudinal and interventional studies, particularly those that test microbiome modulation strategies (e.g., FMT, synbiotics) in MASLD/MASH. As a result, the ability to infer causality or therapeutic relevance remains limited. Lastly, we acknowledge the absence of formal risk-of-bias assessment using validated tools such as RoB 2 or ROBINS-I, and the lack of protocol registration on platforms such as PROSPERO. While we followed PRISMA 2020 guidelines to ensure transparency, these omissions may limit the reproducibility and critical appraisal of the review.

## Conclusion and future directions

This systematic review highlights the significant role of gut microbiome dysbiosis in MASLD/MASH progression. Animal and human studies consistently demonstrate associations between microbial imbalances, metabolite alterations, and liver inflammation and fibrosis. However, future research must address gaps in longitudinal data, standardization of microbiome analysis, and clinical validation of microbiome-targeted therapies. Precision microbiome interventions hold promise, but robust trials are essential for their successful clinical translation.

Advances in high-resolution microbiome analysis – including metagenomics, metabolomics, and integrated multi-omics approaches – have provided valuable insights, yet their translation to clinical practice is hampered by a lack of standardized methodologies and limited longitudinal human studies. Future research should prioritize large-scale, longitudinal cohort studies to clarify microbiome dynamics over time, interventional trials to test microbiome-targeted therapies, and mechanistic studies to establish causality. Moreover, there is a pressing need for the development of validated, non-invasive biomarkers derived from microbiome profiles to aid early diagnosis and disease staging.

Emerging therapies such as probiotics, prebiotics, synbiotics, fecal microbiota transplantation, and precision dietary interventions hold considerable promise. However, their clinical application requires rigorously designed trials with long-term follow-up to assess efficacy, safety, and sustainability. Personalized therapeutic strategies, tailored to individual genetic predispositions, environmental exposures, and microbiome compositions, represent a crucial frontier for future research.

Finally, addressing MASLD and MASH effectively demands a multidisciplinary strategy. Public health initiatives to combat obesity and metabolic syndrome must be integrated with personalized medical interventions targeting the gut-liver axis. By focusing research efforts on these clearly defined priorities, the field can accelerate the translation of microbiome science into meaningful clinical outcomes, ultimately improving patient care and reducing the growing global burden of liver disease.
